# Self-Insured Employer Health Benefits Strategy Established a Negative Cost Trend While Improving Performance

**DOI:** 10.1089/pop.2018.0184

**Published:** 2019-12-02

**Authors:** Steven E. Goldberg, Maren S. Fragala, Jay G. Wohlgemuth

**Affiliations:** Quest Diagnostics, Secaucus, New Jersey.

**Keywords:** employee health plan, employer health care, reduce health care costs, Triple Aim

## Abstract

This case study describes the collaboration between a self-insured employee benefits team and a national health insurance provider to control costs while maintaining program quality and promoting population health. In 2015, Quest Diagnostics well exceeded the full-year expense target for their ∼60,000-life Group Health Insurance (GHI) program. Through proactive changes, physician executive leadership, health plan collaboration, disease-specific population health initiatives, and plan design, Quest GHI annual employer health care cost trend subsequently improved from a year-over-year trend of 5.7% for 2014 to 2015, to 4.6% for 2015 to 2016, to −1.0% for 2016 to 2017, and most recently, 0.3% for 2017 to 2018. The actuarial value of the GHI plan did not decline, and employee cost share also remained unchanged in 2017 and 2018 versus 2016 for the high-performance network option. There was a 3% premium increase for the Preferred Provider Organization option in 2018. A third-party analysis for full year 2017 showed Quest GHI to be 11% more efficient than the mean GHI for programs with a comparable benefit and employee contribution. Early results in 2018 show improvements in the health status of the health plan membership. This article describes an approach for self-insured employers to proactively collaborate with a health plan and pharmacy benefits manager to practice the Triple Aim of improving the patient health care experience and population health while reducing per capita health care spending.

## Introduction

In the United States, more than 178 million (56%) Americans receive insurance coverage through an employer; of these, approximately 60% have insurance from a self-insured employer.^1^ Health care costs (medical and pharmacy) for employers and employees continue to increase at a 6% predicted rate in 2019, on top of the 5%–6% annual increases observed since 2014.^2^ The increase in health care spending has been directly attributed to increased prices for health care services,^3^ while utilization has remained somewhat consistent.^4^ From 2012 to 2016, the largest cumulative increase in spending was for prescription drugs (27.2%), followed by outpatient services (17.1%), professional services (11.2%), and inpatient services (8.3%).^3^ Such widespread continued escalation in employee health care costs is associated with barriers to accessing care, accessing medications, and treatment adherence, as well as other challenges for employees and their spouses, partners, and dependents. Accordingly, self-insured employers need to obtain additional value to offset the trend of higher health care costs.

## Self-Insured Employers: Risks and Benefits

Companies with a self-insured strategy take on the risk for coverage of medical and pharmacy costs for their employees. As health care costs change, these companies assume the burden or benefit. An important benefit is that self-insured companies pay claims as they present rather than paying a fixed “fully-insured” rate that would include an approximately 2%–3% catastrophic premium. Other benefits of self-insurance include (per Kaiser Permanente^5^):

- Greater control over plan design and reporting
- Increased transparency of claims data
- Cash flow benefits
- Reduced premium taxes
- State mandated benefits may be avoided
- Reduced risk charge
- Savings from “unused” plan costs retained by plan sponsor

Since 2005, employers have increasingly offered consumer-directed health plans (CDHPs) that include Health Savings Accounts or Health Reimbursement Accounts (HRAs) and high annual deductibles.^6^ In 2018, 70% of large employers offered a high-deductible option, 39% offered the high-deductible plan as the only option (per the National Business Group on Health), and 29% of covered employees are enrolled in a high-deductible health plan with a health savings option.^6–8^ Although some evidence suggests cost savings from CDHPs,^9^ these plans do not appear to be effective in reducing spending on low-value services,^10^ and they are not the only factor in bending the cost curve.^11^

This article describes how a self-insured employer applied the Triple Aim^12^ (ie, better experience of care, better population health, a negative per capita cost trend) as a management strategy to directly and proactively engage collaboratively with a national health plan (Aetna) and pharmacy benefits manager to achieve a subsequent year-over-year reduction in employer and employee annual cost trend without compromising quality and access to care. The intent of this article is to describe insights into a novel and successful approach to benefit other self-insured employers encountering similar challenges.

## A Self-Insured Employer Strategy: The Quest Diagnostics Experience

Quest Diagnostics is a medical diagnostic and information services company with 45,000 employees in 49 states. The company has multiple work sites throughout the United States, including 23 sites with between 400 to 2000 employees. Quest's health plan membership consists of 60,000 lives (approximately 30,000 employees and 30,000 dependents, spouses, and partners). About 20% of employees work remotely (outside of a main laboratory or operational facility). Approximately 20% of dependents are children younger than 18 years of age. The annual total employer and employee health benefits spend considers 4 components: (1) employer contributions to claims, (2) employer contributions to an HRA, (3) employee biweekly contributions to the premium, and (4) employee out-of-pocket expenses (eg, co-pays, coinsurance).

Quest's results are benchmarked against a Book of Business (BOB) of matched health care companies to put performance into the context of a reasonable standard. Relative to Quest's national health plan BOB, the Quest insured population has a slightly older mean age (35.1 years, versus 33.8 years for the national health plan), a higher proportion of females (57%, versus 51% for the national health plan), and a higher demographic risk based on older age (1.02, versus 0.96 for the national health plan) (fiscal year 2017). Quest also benefits from the relatively long tenure (average of >8 years) of its employees.

### The problem

A performance review in the late fourth quarter of 2015 indicated that the company substantially underestimated full-year health care expenses. Contributing factors included the emergence of a single-source curative treatment for hepatitis C virus infection in the fourth quarter of 2014 and an unexpectedly high number of catastrophic claims expenses (>$1 million/event) during 2015. With a primary focus on bending the annual cost trend, an initial analysis by the employee health plan (EHP) team yielded 4 key observations:

(1) Employee frustration with the cost of the benefit and the benefit experience (eg, process for incorporating funds from a member's HRA into claims adjudication);
(2) Lack of transparency of in and out-of-network providers and significant cost variance;
(3) A majority of expenditures (74%) were concentrated in a small percentage of members (10%). This last observation is common^13,14^ and described as the Pareto principle.
(4) The health status of a certain segment of the at-risk population was not improving (as assessed by both retrospective and prospective measures of morbidity and utilization potential).

Moreover, a third party identified approximately $35 million dollars in annual avoidable emergency room and hospitalization expenses linked to chronic disease (ie, heart failure, coronary artery disease, chronic obstructive pulmonary disease, diabetes, hypertension, obesity, back and neck pain, asthma) and 6 common mental health diagnoses (ie, depression, anxiety, bipolar disorder, eating disorders, post-traumatic stress disorder, substance abuse) (Table 1).

**Table 1.** Cost Savings Opportunity for Employee Population with Improved Chronic Disease Control^*^

*Inpatient (IP) and emergency room (ER) total paid, 10/1/2015–9/30/2016*					
*Condition*	*Persons*	*IP paid, $*	*ER paid, $*	*Total IP and ER paid, $*	*IP ER PPPY, $*
Mental Health-6^**^	5885	11,431,859	880,312	12,312,171	2092
Heart Failure	198	4,041,760	65,102	4,106,862	20,742
Coronary Artery Disease	604	2,416,200	87,435	2,503,635	4145
COPD	184	668,347	19,283	687,630	3737
Diabetes	3201	2,985,992	274,311	3,260,303	1019
Hypertension	4849	5,026,224	369,602	5,395,826	1113
Obesity	1634	1,735,978	116,313	1,852,291	1134
Back and Neck Pain	2819	2,344,588	225,209	2,569,797	912
Asthma	1646	1,454,476	111,682	1,566,158	951
**Total**	**21,020**	**32,105,424**	**2,149,249**	**34,254,673**	**1630**

^*^ Total extrapolated potential savings from care management of 8 chronic conditions in all employees and dependents with variable participation rates.

^**^ The 6 mental health conditions are depression, anxiety, bipolar disorder, eating disorder, post-traumatic stress disorder, and substance abuse.

COPD, chronic obstructive pulmonary disease; PPPY, per patient per year.

Annual reviews were performed to develop strategic approaches to secure better value while maintaining a competitive benefit. In 2015, the company implemented a full replacement CDHP, based on annual assessment to deliver higher value and competitive benefit. Preventive services were covered for the entire value of services without a deductible and reflected the US Preventative Services Task Force's A & B recommendations.^15^ Deductibles and out-of-pocket limits remained consistent between 2015 and 2017, ranging from $750 to $2000 per employee or $1500 to $4000 per family for co-pay select to basic plans, respectively. Out-of-pocket limits remained at $4500 per person or $9000 maximum per family for co-pay select plans and $6350 per employee or $12,700 per family for the basic plan. Employees were eligible for HRAs ranging from $400 to $2000 annually based on coverage level selection and base salary. Between 2015 and 2017, the company implemented additional plan design strategies intended to drive better value without creating barriers to accessing care, including:

- An incentive to use in-network care and implement controls on out-of-network reimbursement
- An incentive to use centers of excellence in preference to any in-network resource
- An incentive to select a narrow network or exclusive provider organization product
- Formulary changes that encouraged use of generics and narrowed the list of brand drugs
- A spousal surcharge to be paid if a spouse on the plan had access to other employer-sponsored health coverage
- Transitioning a select population of terminated employees to the public exchange with subsidy support, thereby avoiding the need for the employee to elect Consolidated Omnibus Budget Reconciliation Act (COBRA) coverage
- Designs that minimize waste for certain episodes of care:
  —Required a second opinion for preference-sensitive procedures (eg, back surgery)
  —Specialty benefit design that creates an incentive to receive specialty medications in a lowest cost setting of care when multiple settings are clinically equivalent

### Approach

Beginning in the second quarter of 2016, governance of the EHP moved from human resources to the chief medical officer, with line responsibility assigned to a physician executive. Program analysis by review of annual national health plan performance reports, de-identified health and pharmacy claims data (2013 through 2017), and longitudinal data from the company's annual health and wellness program identified year-over-year trends in the population tested. Unfavorable movement was identified in several condition cohorts, including obesity, metabolic syndrome, prediabetes, diabetes, hypertension, musculoskeletal conditions, and cancer.

### Triple Aim as a guiding concept

To drive improvements in satisfaction with the benefit and employee experience of care, the team held a series of employee “lunch and learns” during the 2016 open enrollment interval, elevated visibility of the employee feedback portal, and completed targeted “voice of the customer” surveys. Approximately 3000 of Quest's 45,000 employees were directly engaged.

With the Triple Aim^12^ as a guiding concept, Quest's priority was to reduce the annual cost of health care without reducing access to care or adversely affecting clinical outcomes. Cost savings would be redeployed to employee engagement, individual and population health improvement, and sustainability of the benefit.

Two traditional strategies to drive the cost value of annual health benefits also were considered: plan design and health/well-being/population health solutions. Analysis of both health plan standard reports, and a direct review of population health data, identified a potential third strategy: proactive collaboration between the self-insured employer and a national health plan. Overall, the collaborative review suggested opportunity in 4 areas: (1) fraud, waste, and abuse (FWA), (2) utilization management (UM), (3) demand management and identification, and (4) engagement of high-cost conditions and claims.

### Health care fraud, waste, and abuse

Fraud (an intentional deception or misrepresentation) is done with the knowledge that the deception could result in unauthorized benefit to the person committing the fraud or to an intended other person.^16^ The most impactful case of potential fraud Quest encountered involved plastic surgery procedures performed by nonparticipating providers under representation as an emergency. Potential cases were identified through a manual process involving review of case descriptions that excluded any member identifying information.

Waste is the overutilization of services (not caused by criminally negligent actions) and the misuse of resources.^17^ The most impactful cases of potential waste Quest encountered involved hospital claim submissions for a given episode of care that were not supported by the clinical history, clinical record, or involved an outlier level of claims expense. Claims from out-of-network or nonparticipating facilities proved most challenging. The employer team alerted the health plan to the opportunity to improve diligence in identifying such claims prior to payment. The teams collaborated on solutions to better mitigate out-of-network occurrences and associated elevated costs when a high-quality in-network service was available. The collaborating national health plan was highly responsive.

The term abuse refers to practices that are inconsistent with sound fiscal, business, or medical practices, and that result in unnecessary cost or reimbursement for services that are not medically necessary or that fail to meet professionally recognized standards for health care.^16^ The single largest opportunity Quest identified involved the repetitive use of advanced transportation services for circumstances that, upon direct member evaluation, did not meet medical necessity criteria and were deemed to be not clinically indicated.

### High-cost conditions/high-cost claims

**FIG. 1.**
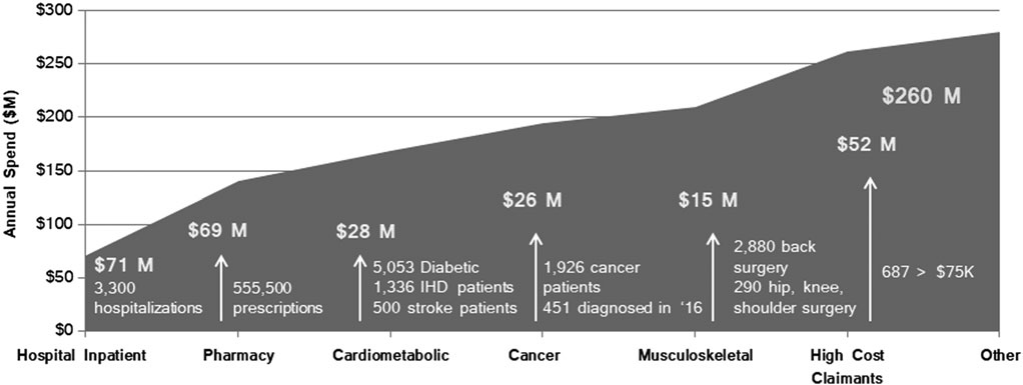
Key spend categories of the insured employee population. IHD, ischemic heart disease.

Overall, for self-insured employers, approximately 1%–2% of members drive 30%–35% of annual claims with an average claims cost of $122,000.^6^ Quest's analysis showed that 10% of its health plan membership was responsible for 72% of claims spend in 2015. High-cost claims (≥$75,000) represented 42% of Quest's national health plan claims in 2015, 41% in 2016, and 38% in 2017. The pattern of the distribution of employer spend is shown in Figure 1 and incurred medical and pharmacy claims for 8 chronic conditions are shown in Table 2.

**Table 2.** Allowed and Paid Medical and Pharmacy Claims for 8 Chronic Conditions in 2017

	*Allowed*		*Paid*	
	*Annual Total ($)*	*PPPY ($)*	*Annual Total ($)*	*PPPY ($)*
Heart failure	1,028,591	2765	875,462	2353
Coronary artery disease	9,417,928	4401	8,097,603	3784
Chronic obstructive pulmonary disease	2,226,070	3511	1,884,125	2972
Diabetes	16,993,456	4168	13,833,672	3393
Hypertension	7,285,998	728	4,485,222	448
Obesity	5,347,907	1007	4,338,108	817
Back and neck pain	12,344,416	2082	9,166,947	1546
Asthma	5,590,847	1435	4,259,321	1094
TOTAL for 8 conditions in 2017	60,235,212	2838	46,940,461	2212
TOTAL in 2017^*^	342,040,911	5947	271,381,122	4718

Direct medical (professional, inpatient, and outpatient) and pharmacy costs from episode treatment groups (ETG Base, Optum Symmetry Grouper) incurred during the 2017 calendar year.

^*^ TOTAL in 2017 spend is based on original claims.

PPPY, per patient per year.

The teams were aligned on the premise that early identification, engagement, and direction of members with potential high-cost conditions to settings of best care at best value would contribute to improved outcomes and reduce avoidable costs. In Quest Diagnostics' experience, solutions must be organized by disease state and incorporate multiple elements, including early identification, engagement, correct diagnosis, care pathways by episode that track to best evidenced-based care, care delivery in the lowest cost setting, and continuity of care facilitated by an appropriately situated provider supported by health plan care management staff resources. BOB data from Quest's national health plan indicated that the annual per member per month (PMPM) cost trend in high-cost claim cases well exceeded the PMPM BOB trend of 3.5% (2015), 5.5% (2016), and 5.0% in 2017. The high-cost claimant trends of ≥$75,000 per member per year (PMPY) were 11% and 10% in 2016 and 2017, respectively; and those ≥$300,000 PMPY were 16% and 16% in 2016 and 2017, respectively (personal communication; Paul C. Mendelowitz, MD, MPH; October 9, 2018). In sum, the data indicate that an employer is less likely to mitigate overall annual cost trend without an intentional high-cost condition/high-cost claim strategy.

### Utilization management

Self-insured employers delegate oversight of clinical services and financial transitions within the health care ecosystem to either a health plan or a third-party administrator. Quest's national health plan demonstrated thoughtful, sound strategies to appropriately manage demand, review processes to ensure medical necessity, support clinical efficiency across the continuum of care, and review claims for clinical and financial accuracy – all without impeding access to care. Quest's experience indicated that a regular cadence of direct dialogue (ie, face-to-face meetings between the self-insured employer group health insurance team and the national health plan designated clinical team) that focused on process and outcome metrics of the implemented care model resulted in improved execution of some processes on Quest's behalf.

### Implementing the collaboration

Most self-insured employers and health plans interact 1–2 times per year during the end-of-year review and at time of discussion of renewal costs. Establishing a cadence of initial biweekly, and eventually monthly, dialogue between a self-insured employer and its primary health plan was a new undertaking for both organizations. The parties had to make decisions regarding the agenda, meeting participants, dashboards, data needed to inform the dialogue, goals, metrics, and actions needed to drive objectives. The health plan provided additional subject matter experts (eg, digital engagement, behavioral health) as needed. The Triple Aim^12^ was the organizational basis for 3 areas of primary focus.

- The highest priority was cost trends and identified priorities in FWA, UM, demand management, and high-cost conditions/claims. The team reviewed established high-cost claims, high-cost conditions at risk for high-cost claims, and short-stay diagnoses. Attention was directed to both care coordination and care efficiency opportunities. The national plan's care model leveraged a spectrum of clinicians for this engagement.
- The second focus area was population health, with discussions organized by condition or disease state. Initial attention was on cardiometabolic, cancer, and musculoskeletal categories.
- The third priority was a focus on barriers to employee engagement and a positive experience of care. The health plan was the primary source of these materials, and the Quest Diagnostics analytics team ran ad hoc queries to inform activities. Proactive monitoring and engagement of the health plan by the Quest Diagnostics EHP team benefited from contributions by the lead of health benefits, workplace health promotion, and data scientist. Monthly reporting included financial results.

## Results

The Quest Diagnostics Group Health Insurance (GHI) annual health care cost trend improved from a year-over-year trend of 5.7% from 2014 to 2015, to 4.6% from 2015 to 2016, to a decline of 1.0% from 2016 to 2017, while holding employee contributions flat for 2017 and maintaining a comparable benefit and member cost shared with the pool. Quest's full year 2018 employer health care cost trend was measured at 0.3%. To benchmark this experience, Quest Diagnostics participated in a third-party claims analysis for full year 2017. The plan costs for the 60,000 member GHI program were compared to a database representing 1 million lives by a third party (unpublished data; Michael Tessler, FSA, MAAA and Michael Perlmutter, MBA [Willis Towers Watson's 2018 Financial Benchmark Survey]; June 11, 2018). That analysis found that for full year 2017, the Quest Diagnostics program was 11% more efficient than the mean for a comparable benefit. Quest Diagnostics plan costs also were assessed as being 1% more efficient than the top quarter of the study pool. Efficiency was determined by evaluating health care expenses on an equivalent basis, after adjusting for demographics, geography, and plan value.

### Key indicators 2016–2017

**FIG. 2.**
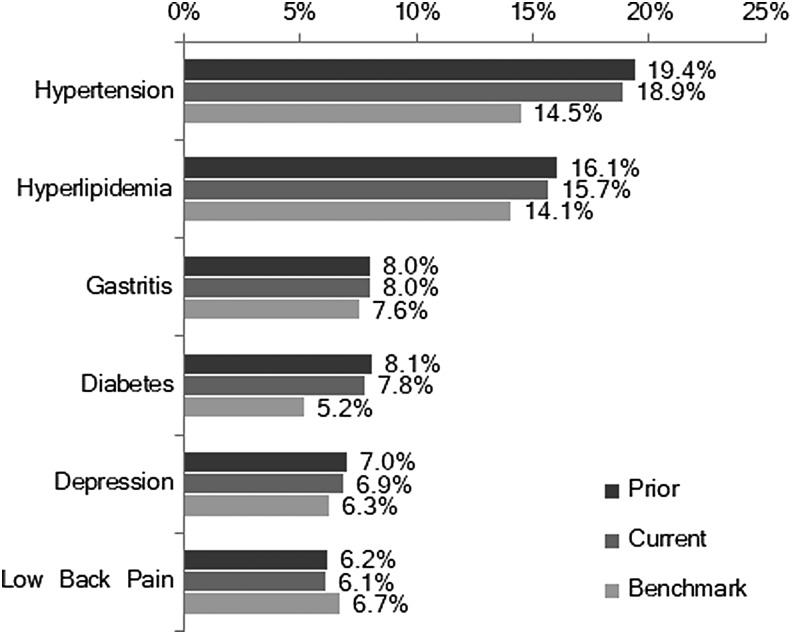
Population prevalence of chronic disease.

According to Quest's national health plan, primary drivers of the improved Quest Diagnostics claims trend were attributable to improvements in musculoskeletal, injury/poisoning, digestive, and respiratory conditions. Oncology diagnoses remained the primary driver of high-cost claims. Quest's cancer prevalence rate increased year over year, but prevalence remained below the BOB for the national health plan. Approximately 40% of Quest's oncology cases were patients with multiple or advanced cancers, which was unchanged from the previous year. The prevalence of musculoskeletal conditions was stable, but related spending decreased from 2016 primarily because of a drop in large claimant costs. Pregnancy-related claims were within range of benchmarking with ongoing strategies focused on engaging expectant mothers with nurse resources and education to support healthy, full-term pregnancies. The majority of high-cost claimants had chronic, potentially “impactable” conditions (Fig. 2).

### Quest Diagnostics preventive screening results 2016–2017

During this interval Quest focused on driving each element of the Triple Aim. The team actively sought confirmation of continued progress on measures of the experience of care and population health while improving the cost trend. The age of Quest's employees was the most dominant factor affecting increasing prevalence of disease compared to prior years. In measures of participation in preventive screening, Quest experienced improved rates for colorectal cancer screening, cervical cancer screening, child preventive visits, and adult preventive visits. Reductions were experienced in rates for mammography (1.5%), child immunization younger than age 2 years (0.6%), and well-baby preventive visits younger than age 2 years (0.4%). Reductions in mammography screening rates track population changes and follow revised breast cancer screening guidelines released by the US Preventive Services Task Force in 2009.^18^ Declines in childhood immunizations and preventive visits may follow medical, religious, philosophical, or socioeconomic reasons.^19^ In addition, the employee assistance program offers all employees and dependents 6 counseling sessions at no cost per year. Engagement in this program is above BOB comparators at 8.0% in 2017, with 68.2% of clinical cases resolved within the program.

### Quest Diagnostics population health status 2016–2017 - ActiveHealth Index

The ActiveHealth Index (AHI) comprises a series of measures previously developed by Quest's national health plan to measure population health status and to gauge the impact of the population health interventions. These measures captured multiple dimensions of health. Clinical knowledge and composite data are derived from a variety of sources (eg, medical and pharmacy claims, laboratory test results, health assessments, biometrics, electronic medical records) and can be factored into health metrics for a data-driven approach to population health management. AHI provides a single measure of health status based on 10 dimensions of health, including 4 established dimensions (ie, age and sex, behavioral health conditions, medical conditions, geography) and 6 “impactable” dimensions (ie, evidence-based medicine, lifestyle and biometric risks, medication adherence, preventive care, at risk for conditions, self-perception of health). The impactable dimensions can be reflected as the percentage of “ideal” health the population has achieved (Impactable Health Index). The gap between the Impactable Health Index and 100 yields the Health Improvement Opportunity.

**FIG. 3.**
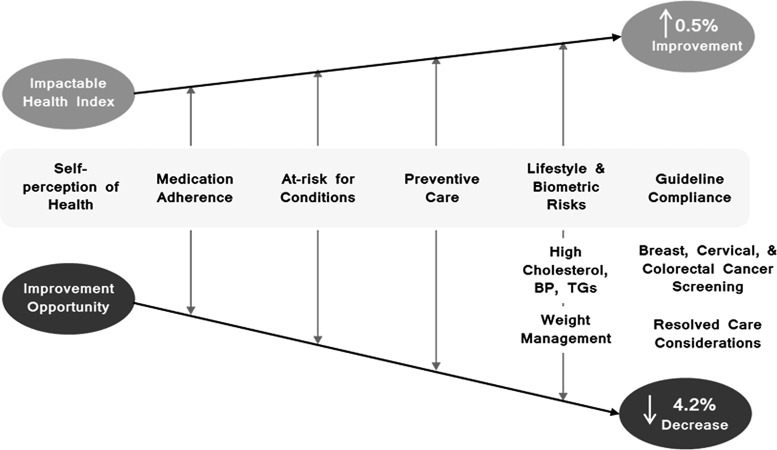
Population health status reflected by dimensions of the Active Health Index in 2017. BP, blood pressure, TG, triglycerides.

Between 2016 and 2017, the Impactable Health Index improved by 0.5% and the Health Improvement Opportunity decreased by 4.2% (Fig. 3). Declining opportunity is indicative of an improvement in the population health and/or the care activities that have taken place to close gaps in care. The improved population health status was attributed to improved compliance with national health plan care gap communications to both members and their physicians in the following areas: biometrics (high cholesterol, blood pressure, triglycerides, and overweight status), condition/drug monitoring, adding/intensifying medical therapy, and preventive care screening for breast, cervical, and colorectal cancers.

In sum, attention to reducing the cost trend did not increase barriers to access to care or adversely affect the health status of the employee population.

### Advancing performance on the other two elements of the Triple Aim

In establishing processes via collaboration with its health plans, Quest Diagnostics has positioned the company and its employees to extract better financial value from the health care system. Continued improvements in the experience of care of an individual employee and improving the overall health of the health benefit plan (HBP) population are critical elements to the sustainability of the benefit. Between 2018 and 2016, Quest redeployed funds from cost savings and methodically made additional, incremental investments to affect metrics in these areas.

Regular annual screening and subsequent analysis enabled the team to identify the conditions and disease states responsible for the majority of the Quest Diagnostics spend and the employee sites of greatest challenge. It is Quest's experience that these data help inform optimal “upstream” engagement for GHI members and the company. Employee participation in the annual health screening event has remained high (+80% in 2017 versus a national average participation rate of 40%) for similar rewards-based programs.^20^

Quest recently added Physician Health Information Sessions to its annual employee wellness screening program, whereby participants can speak with a physician to better understand their results, prepare for a shared decision-making session, and be referred to a network provider for definitive care. Participant feedback indicated that the information sessions improved participants' understanding of results and perceived relevance of testing (94%). Most participants were satisfied with the sessions (93%), felt that the sessions were personalized (94%), and would participate in the sessions again (94%).

The national health plan vendor implemented new auto-verification technology to improve Quest's members' experience of the operation of the HRA. Satisfaction with the telephonic advocacy service remains high (85% highly satisfied) for members using the service for a wide range of activities, from benefit questions to assistance securing an in-network provider.

Grand Rounds was implemented in July 2016 to assist members requesting a second opinion for diagnosis and/or treatment or to identify a high-quality, in-network provider within their local geography. Utilization of this service (for either a telephonic second opinion or a provider encounter) averages >30 services per month (range 15–100). The objective is to assist providers in the diagnosis and treatment of complex clinical cases, and to share information regarding shared decision making for preference-sensitive procedures.

In June 2017, Rx Savings Solutions was introduced to serve as a trusted prescription drug advocate for members, and to target members who may be able to save on prescriptions based on their present regimen and its variance from the implemented formulary and price savings opportunities within the retail network. The company also serves as a partner to Quest's EHP to inform discussions on formulary selection/design with Quest's pharmacy benefits manager, such as identifying high-cost but low-value drugs that can safely be excluded. Approximately 25% of employees have enrolled in the service, and the overwhelming majority (>80%) who are engaged express high satisfaction with the experience and outcome of the service. Other solutions have been implemented to engage particular cohorts, including type 2 diabetes prevention (Omada), diabetes management (OnDemand), and tobacco cessation (QuitforLife), with 10%–33% of those eligible enrolled in year 1. Formal evaluation of experience with those programs is pending.

## Discussion

Quest Diagnostics has achieved a reduction in the year-over-year cost trend of its GHI program. Over a 24-month period, the Quest Diagnostics GHI annual health care cost trend improved from a year-over-year PMPM claims trend of 5.7% from 2014 to 2015, to 4.6% from 2015 to 2016, to negative 1.0% from 2016 to 2017, while holding employee contributions flat for 2017. It was the first year in the past 10 years that Quest Diagnostics achieved a PMPY reduction in both employer and employee full-year health care costs. Based on analysis by a third party, for full year 2017, Quest Diagnostics has established a self-insured employee HBP that is 11% more efficient than the mean and 1% more efficient than the upper quartile for a comparable benefit and comparable employee contributions (unpublished data; Michael Tessler, FSA, MAAA and Michael Perlmutter, MBA [Willis Towers Watson's 2018 Financial Benchmark Survey]; June 11, 2018).

Although this case study describes a strategy and associated measures, it is not possible to extract clear proof of a specific impact of any particular strategy on cost reduction. For example, it is not possible to separate the relative impact of plan design from proactive changes (physician leadership, health plan collaboration, disease-specific population health initiatives). However, previously, plan designs in CDHPs have been shown to be ineffective in activating the adoption of productive health behaviors,^21^ yet do provide a supportive environment for those who are more activated to manage their health.^22^

In addition, cost efficiencies in CDHPs appear to be related to younger, healthier workers electively enrolling, and are mitigated when population demographic risk is considered.^21,23^ Moreover, CDHPs had little impact on preventive services, but did impact prescription drug use in the direction of brand to generic.^21^

Despite a proactive strategy, other factors could be responsible for some of the improved performance. It is possible that the full-year 2015 was a single year outlier in terms of the percent of catastrophic claims (>$1 million) and high-cost claims (≥$75,000) when evaluated in the interval of 2012–2018. Quest could have subsequently experienced a regression to the mean. Although 2015 was indeed an outlier in terms of catastrophic claims events, the percentage of dollars attributable to claims costing ≥$75,000 has been consistent within approximately 5% year over year (and between 38%–42% of claims dollars between 2015 to 2017). Other initiatives that could have contributed materially to the reduced trend include (1) the 2016 to 2019 expansion of the percent of members receiving care in an exclusive provider organization (0 to 25%), and (2) the move of a select number of members to the public exchange with subsidy support. Regardless, in numerous case examples the model implemented achieved a reduction in appropriate avoidable claims expense following careful high-cost claims reviews. Finally, approximately 1200 more individuals/employees elected the Quest Diagnostics EHP in 2018 versus 2017, reflecting positive member experience.

A critical appraisal of the practice of appointing physicians to serve as leaders of health benefits within a self-insured employer warrants additional study.

## Conclusion

Cost trends remain a significant barrier to the sustainability of a self-insured employer health benefit. Adopting the novel approach of proactive collaboration between self-insured employer and health plan, and prioritization of opportunity described herein, may enable other self-insured employers to achieve similar success in terms of increasing efficiency and achieving the Triple Aim.
